# The Contribution of Chaplaincy to Primary and Community Care: A Semi-Structured Interview Study With Clients

**DOI:** 10.1177/21501319251357528

**Published:** 2025-07-17

**Authors:** Annelieke Damen, Carmen Schuhmann, X.J.S. Rosie, Marjo van Zundert, Gaby Jacobs, Hanneke Muthert, Erik Olsman, Anja Visser

**Affiliations:** 1 University of Humanistic Studies, Utrecht, The Netherlands; 2 University of Groningen, Groningen, The Netherlands; 3 Maastricht University, Maastricht, The Netherlands; 4 Protestant Theological University, Utrecht, The Netherlands

**Keywords:** chaplaincy, spiritual care, spiritual needs, chaplaincy goals, chaplaincy outcomes

## Abstract

**Introduction:**

A broad range of studies have associated spirituality with health outcomes. However, the integration of spiritual care in primary and community care has substantially lagged behind. Chaplains, as specialist spiritual caregivers, are increasingly employed in primary and community care to fill the gap. To investigate the implementation of chaplains in these settings from the perspective of clients, this study focused on the following research question: what are primary and community care clients’ reasons to seek chaplaincy care, their ideas of care goals, and what outcomes of care do they report?

**Methods:**

24 Dutch chaplaincy clients were interviewed.

**Results:**

Clients sought support from a chaplain for existential concerns, or an existential struggle encompassing several areas of life. They described goals and outcomes of care in 3 domains: (1) the relationship with the chaplain, which included being seen, heard and acknowledged; (2) meaning-making, where they gained insight into and/or processed life-events, and connected more with themselves, others and/or the sacred; and (3) well-being, which included feeling better and finding peace.

**Conclusions:**

This study provides novel insights into clients perspective on chaplains’ contributions in primary and community care. Their experiences are key to further shaping the implementation of chaplaincy in these settings.

## Introduction

Over the past decades, increasing attention has been given to the relation between spirituality and health.^1-3^ Here, spirituality is understood as a universal human phenomenon, referring to the way in which people search for meaning, purpose, and connection.^4^ Spirituality may be related to but does not coincide with religion, as people, when searching for meaning, may draw from religious and non-religious meaning frameworks.^5^ Spirituality has been associated with better coping with a wide range of illnesses or a variety of stressful situations.^2^ Conversely, numerous studies have associated a loss of meaning, for example in the face of illness, death, loss or other major life events, with higher levels of distress such as anxiety, depression, PTSD symptoms, and poorer physical health.^6-8^ Finally, a broad range of studies have associated spirituality with health behaviors and lifestyle choices.^2,9^

Given the connection between spirituality and health, holistic approaches to health care which recognize spiritual care as a crucial dimension of care provision have gained influence in Western societies.^4,10^ Healthcare chaplains are increasingly seen as integrated members of interdisciplinary healthcare teams.^11,12^ As specialist spiritual caregivers, chaplains support people in their search for a renewed sense of meaning.^4,13^ Historically, chaplaincy care has referred to care offered within Christian and Jewish traditions.^14^ Over time, the term has come to designate care in the context of other worldview traditions too, among others Muslim, Buddhist, Hindu, Pagan, and humanist traditions. Healthcare chaplaincy is transforming into a denominational and non-denominational profession, with chaplains not just caring for patients of their own worldview tradition, but for all patients irrespective of their worldview.^15^

In the settings of primary and community care, the engagement with spirituality has lagged behind inpatient care contexts.^16^ While in the past chaplains mainly cared for patients within healthcare institutions, chaplains are now increasingly becoming caregivers in primary and community care^17,18^ following the deinstitutionalization of different healthcare domains and the development toward more outpatient and non-residential care in the Global North.^19-21^ For example, chaplains started to work in outpatient clinics, outpatient palliative care, alongside general practitioners, and in The Netherlands in Centers for Life Questions (CLQ) where the general public can contact a chaplain for a home visit.^16-18,22,23^ Research among oncology outpatients in the United States (US) and Germany indicates that approximately 30% to 90% indicate at least one religious/spiritual (r/s) concern^24-26^ Moreover, up to 83% of outpatients in the Global North want their general practitioners to speak with them about their r/s beliefs,^27,28^ and/or appreciate a referral to a chaplain.^22,26,29^

According to the available research in primary and community care in the US and Scotland, patients seek chaplaincy support because they want to share their personal stories, receive support around major life events (e.g., bereavement, relationship breakdown, family breakdown, depression, loss of self-confidence and identity), receive support around medical decisions, discuss their spiritual/religious questions, and participate in religious practices.^22,30^ Furthermore, studies indicate various outcomes of chaplaincy care, like higher (spiritual) wellbeing, higher health-related quality of life, and better religious and general coping.^16,23,30-34^ Nevertheless, research into spiritual care in the context of primary and community care is still scarce: “Outpatient chaplaincy is a new specialty in healthcare, with a relative paucity of research studies exploring the need for spiritual care interventions in ambulatory settings” (p. 81).^22^ This specifically holds true for research into the perspective of patients on chaplaincy in these settings.

The present study seeks to expand what is known about chaplaincy in primary and community care by focusing on the perspective of chaplaincy patients. As they are usually called clients rather than patients by Dutch chaplains, we will use these terms interchangeably. The study addresses the following research question: what are primary and community care clients’ reasons to seek chaplaincy care, their ideas of care goals, and what outcomes of care do they report? In the study, various client groups participated. Exploring, describing, and understanding their experiences is key to further shaping the implementation of chaplaincy in primary and community care.

## Materials and Methods

### Qualitative Approach, Research Paradigm, and Positionality Statement

For this study the researchers employed a narrative research approach, focusing on the collection of stories that people construct to make sense of their lived experiences.^35^ The study was conducted under a constructivist/interpretative research paradigm, that included considering reality as a subjective construction.^36^ The research was conducted in a collaboration between the University of Humanistic Studies, the University of Groningen and the Tilburg University. All 3 Dutch universities have research programs aimed at understanding processes of meaning-making and humanization. All authors are academically educated researchers working in the field of chaplaincy studies. Their backgrounds are humanist, Christian and unaffiliated, and they have in-depth knowledge of the different chaplaincy fields in which the respondents were recruited. The first author AD and third author XR conducted the interviews with the respondents. The greatest sociocultural differences between them and the participants comprised their gender, age, educational level, and worldview background. Based on both researchers’ experiences with chaplaincy, these differences were partially mitigated through a common understanding of what chaplains do. However, the different positionalities also challenged AD and XR to find the right language to speak about meaning-making.

### Participants

The study was carried out in the context of a nationwide research project in the Netherlands, the *Knowledge Workplace on Meaning and Spiritual Care (Kenniswerkplaats Zingeving)*. The wider aim of this project was to professionalize the field of chaplaincy care and the dimension of spiritual care in primary and community care settings. Respondents were recruited through chaplains who participated in learning networks in this project. Chaplains worked with different types of Dutch clients. These comprised 50-plus year olds, veterans, victims of earthquakes due to gas extraction in the North of the Netherlands, unhoused people, and mental health patients. We have differentiated these types of clients in the analysis, because their access to chaplaincy care is organized through different financial models, influencing the possible duration of their care.^i^

Respondents were included when they received primary and community care, were 18 years or older, and within 3 months of the end of the chaplaincy support (or during a calmer period of the support). Respondents receiving care from a psychiatrist were excluded. The sociodemographic characteristics of the 24 respondents are reported in Table 1. The sample (50% female; average age, 61; 50% middle school educational level) considered themselves Christian (42%) and “nones” (54%). This approaches the religious landscape of the general Dutch population with 34.2% Christians and 55.4% nones.^37^

**Table 1.** Sociodemographic Characteristics (N = 24).

Variables	N (%)
Age in years, mean (SD; range)	61 (13.2; 49-95)
Gender	
Male	12 (50)
Female	12 (50)
Educational level (N = 22)	
Middle school	11 (50)
High school/some college	3 (14)
University or postgraduate degree	8 (36)
Denomination	
Christian (Protestant, Roman Catholic, Christian)	10 (42)
Not affiliated	7 (29)
Spiritual	4 (17)
Humanist	2 (8)
Buddhist	1 (4)
Chaplaincy working areas	
50-plus year old	7 (29)
Veterans	5 (21)
Victims of gas extraction (earthquake area)	4 (17)
Unhoused	4 (17)
Mental health patients	4 (17)

### Procedure

Chaplains approached eligible clients via mailed invitation letters, over the phone, or face to face. Clients who were interested in participating and who consented that the researchers could approach them, were contacted by AD or XR to plan an interview at their home, another location, or online. The interviews lasted between 30 and 60 min. The interview guide included clients’ spiritual concerns, goals of care, and outcomes formulated in everyday language:

1. the reason why the respondent came into contact with the chaplain,
2. what the respondent hoped would happen because of the support by the chaplain,
3. what (if anything) had changed because of the support by the chaplain.

To be able to put clients’ answers into context, we also briefly asked about:

4. what the chaplain did when providing support,
5. the length of the chaplaincy support in number of conversations and weeks/months,
6. sociodemographic characteristics.

### Analysis

For the analysis of the data, we followed the Qualitative Analysis Guide of Leuven (QUAGOL).^38^ This analytical approach consists of 2 parts, which both include 5 stages. We generally followed the steps of the guide, thus following the directions by Dierckx de Casterlé and colleagues to use the QUAGOL method “as a guiding tool rather than as a strict procedure or technique.”^38^ We carried out the 5 stages of part one, the preparation of the coding process, as follows:

1. The interviews were recorded and transcribed verbatim by AD and XR. Subsequently, AD, CS, and XR thoroughly read the interviews.
2. AD and CS wrote narrative reports to capture the essence of the respondent’s experience in response to the research question. The narrative reports were checked and supplemented by XR.
3. AD and CS translated the narrative reports into abstract concepts: individual conceptual interview schemes.
4. AD compared the individual conceptual interview schemes with the interview data for further adaptation, completion, and refinement.
5. AD and CS developed an overarching conceptual interview scheme based on the individual conceptual interview schemes.

We then moved to part 2, the actual coding process, carrying out the 5 stages in this part as follows:

6. AD entered the overarching conceptual interview scheme in ATLAS.ti.^39^
7. AD coded the interviews with the help of the overarching conceptual interview scheme. Codes were checked by XR.
8. AD, CS, XR, and AV analyzed if every citation fit to the codes, and after refinement, articulated the codes into their own words.
9. This was translated into a meaningful storyline in response to the research question by AD and CS, and checked by XR and AV.
10. Finally, the research team wrote down the results.

### Ethical Approval

The study was approved by the Ethical Review Board of the University of Humanistic Studies, file number 2021-20. Respondents were informed about the study, including their rights as participants, by written letter and orally. They were granted the possibility to ask questions. All the participants signed an informed consent before the start of the interview.

## Results

### Core Findings

From the analysis of our interview data, we can formulate the following core findings in response to our research question:

Outline of the context: Clients were referred to chaplains by caregivers or other people, met them by chance, or approached them on their own accord. Chaplains were the only caregivers clients saw or were complementary to other caregivers. In some cases, the chaplain was the client’s last hope after multiple (failed) other care trajectories. For the 50-plus year old and the mental health clients, chaplaincy support usually comprised a maximum number of 5 contact moments (the maximum number reimbursed by the government). For the veterans, victims of gas extraction and the unhoused people, support was long-term, sometimes up to several years. Here, chaplaincy support was what we call much more “fluid”: it could be intensive for times but also absent for months. The support not only involved fixed appointments, but also (short) moments of contact over the phone. Moreover, the support was broader than existential conversations and also included care in other areas of life.
Reasons for contact: Clients had manifold reasons for seeing a chaplain. Some sought support for one or several existential concerns. Others experienced an intense existential struggle; they described themselves as being in crisis in different areas of life. They had lost trust in themselves, others, and/or the government. For many of the participants, loneliness was a trigger to seek chaplaincy support.
Goals of care: All clients, independent of the gravity of their existential concerns, formulated goals within 3 domains. First, regarding the relationship with the chaplain, clients hoped to find a safe space to tell their story and a conversation partner to spar with. They wanted to be seen, heard, and acknowledged by the chaplain. Second, regarding meaning-making, they hoped to gain insight into themselves, develop a new perspective on life-events, integrate or process life-events and feelings, develop a grip on life, and/or find a future perspective. They also hoped to connect more with themselves, others, and/or the sacred. Third, regarding well-being, they hoped to feel better or find peace.
Experienced outcomes: Clients referred to outcomes within the same domains as their goals of care. First, they reported being seen, heard, and acknowledged in the relationship with the chaplain. They felt (temporarily) less lonely. They had regained (some) trust through trusting the chaplain. Within the domain of meaning-making, they reported having a better understanding of themselves; being able to put life circumstances into perspective; being able to feel and/or let go of emotions; having processed life-events; having regained a grip on life; and seeing a path forward or future perspective. They also reported a regained connection with themselves, others, and/or (sacred) sources of strength. Regarding the third domain, they mentioned increased well-being.
Effective elements: As effective elements that lead to these outcomes, clients mentioned relational qualities of the chaplain, chaplains’ meaning-making competencies, and the safe space of chaplains’ confidentiality.

We will now discuss the above core findings in more detail, illustrated with quotations from the interviews. See for a complete overview of the results Table 2.

**Table 2.** Results of the Study.

Core findings	Domains	Subdomains
Outline of the context	- Up to 5 contact moments - Long-term and “fluid” support	
Reasons for contact	- One or some existential concern(s) - An intense existential struggle	
Goals of care	1. The relationship with the chaplain	1. a safe space to tell their story 2. a conversation partner to spar with 3. be seen, heard, and acknowledged by the chaplain
	2. Meaning-making	1. gain insight into themselves and/or develop another perspective on life-events 2. integrate or process life-events and feelings 3. develop a grip on life, and/or find a future perspective 4. connect more with themselves, others, and/or the sacred
	3. Well-being	1. feel better or find peace
Experienced outcomes	1. The relationship with the chaplain	1. being seen, heard, and acknowledged 2. feeling (temporarily) less lonely 3. having regained (some) trust
	2. Meaning-making	1. gained insight into themselves and/or life circumstances 2. allowed to feel, let go, and/or process emotions and life-events 3. having regained a grip on life 4. seeing a path forward/future perspective 5. having a regained connection with themselves, others, and/or (sacred) sources of strength
	3. Well-being	1. experiencing increased well-being
Effective elements	- Relational qualities of the chaplain - Meaning-making competencies - The safe space of chaplains’ confidentiality	

### Outline of the Context

The 50-plus year-old clients found the chaplains by hearing about them from other people, meeting them in church, seeing an advertisement, or through a referral from a general practitioner or other caregiver. Veterans were referred to chaplains by the mental health department of the military. Mental health clients were referred through the mental health institution. The referrers included social workers, nurses, and psychiatrists. The victims of gas extraction found the chaplain through various ways: through other people, at a protest, through a touring chaplaincy van, or on a website. The unhoused clients met the chaplain through the initiative of the chaplains themselves. They were approached at a shelter or at a church service for the unhoused:

Well, I met her: she just came to sit with me and she heard me talking about, yeah, something, I don’t even remember [. . .]. But she came to sit with me, very open, open face. That also makes a big difference I think, if you smile and are open, because I experience her as an open person who also greets people by name. [Interview X20]

For some clients, the chaplain was the only caregiver they saw. Other clients experienced the chaplain as complimentary to different caregivers. However, in some cases, the chaplain was the client’s last hope after multiple (failed) care trajectories, or the only caregiver left after their treatment had finished:

That was really just perfect, how that matched up. Otherwise, you just sit still for days after such an hour-long treatment, you’re still mentally stuck. But then there was usually a conversation with the chaplain after a mental health treatment, yes, then I could completely unwind. (Interview X9)

In response to the question ‘How do you see my life going forward’, the caregiver remained silent. After a while caregivers gave up: figure it out yourself, [. . .] maybe you should try without care for a while. Well, that’s really not going anywhere. So, the social worker referred me to the chaplain. (Interview X10)

For the 50-plus year old and mental health clients, chaplaincy support usually comprised the maximum number of 5 contact moments reimbursed by the government. For most of them, this was enough, but some indicated that more support would have brought them to another level. For 2 clients, 5 conversations were clearly not enough, there was a mismatch between their existential concerns and the amount of chaplaincy care. For the veterans, victims of gas extraction and the unhoused, the support was long-term and much more “fluid.” Next to fixed appointments, the chaplain and client had contact over the phone or through text messages, for up to several years:

Yeah, there’s a thread there. It’s not like every moment. [. . .] She gets in contact or she comes over [. . .] and we have apple pie and just talk. (Interview X3)

Moreover, the support was broader than conversations, and included leading a funeral service, being a liaison to the municipality, mediating conflicts, supporting with paperwork, conversations with a lawyer, bike tours and beach walks, or visiting meaningful places together such as the former house:

There is a part of the wall of the old house, and we looked at it and touched it. (Interview X5)

### Reasons for Contact

Seven clients mentioned one or several existential concerns. Their reasons for seeking chaplaincy support included an accumulation of sorrowful life-events, fear for one’s own death, or dealing with the illness and/or death of a loved one:

I was at the doctor’s office, and there I discussed that my mother had died, and that I just thought it was very strange and weird that I couldn’t cry about my mother. When a friend or girlfriend had died in the past, I was crying for three days. And I didn’t have that with my mother. Well, I found that very strange. And I talked to the family doctor about that, and the family doctor then referred me to the chaplain. (Interview X24)

Seventeen clients mentioned that they were dealing with an intense existential struggle, the basis of their life had become unsteady or had even fallen away. They experienced crisis in different areas of life, which sometimes had even led to suicide attempts. The crisis arose for example from severe mental health problems, leading to chronic pain, conflicts at work, the loss of work, and/or a divorce. Or they struggled with the (possibility of a) traumatic loss of a home, resulting in financial difficulties, alcohol abuse, and/or conflicts with the government.

We got hurt [. . .] and I was hanging by a thread. (Interview X3)

I say: I feel like I’m all wrapped up in cling film, that plastic, like we do with pallets and stuff, [. . .] you can see through it, but you can’t do anything anymore, you don’t know what to do or say and you can’t get out. And that trapped feeling, and every time there’s something more and there’s something more, just every time something unexpected that you couldn’t have imagined. (Interview X5)

These clients dealt with multiple experiences of loss. They had lost trust in themselves, others, and/or the government. They struggled with strained family relations, a loss of social life, and a loss of a future perspective on life:

Because no matter how you look at it, for the next year and a half to two years, I will still have lost the grip and control over a part of my own life. I feel damaged by the government. I have lost trust in myself and the government, in neighbors, and they in me, perhaps. (Interview X2)

At that time, I was losing a lot [. . .]. That I thought: I don’t know very well how I envision the future for myself. (Interview X14)

Finally, loneliness was for many a trigger to seek chaplaincy support:

I said, “I’m standing here, I’m fucking alone” [cries]. And then you are alone, and it hurts so much. You can’t get rid of it. And I tried to say that to others, but they didn’t listen. (Interview X25)

### Goals of Care

We have structured the goals of care into 3 bigger domains. The first domain is about the relationship between the clients and the chaplain. The second domain is focused on the meaning-making of the client. The third domain comprises the clients’ well-being. Regarding the first domain, clients hoped to find a safe space to tell their story, to speak about what was bothering them. They hoped this would be a space where they could be themselves and have a conversation on the same wavelength. They wanted to be seen, heard, and acknowledged by the chaplain:

I very much wanted to be heard, seen and acknowledged in, well, that it’s just very shitty, the situation we’re in, and that that has eaten up an awful lot of our lives. (Interview X5)

Regarding the second domain of meaning-making, they hoped to gain insight into themselves and life-events, and to develop another perspective on life-events:

I had some things that weren’t going well in my life, and I was hoping that she could untangle them a little bit. (Interview X26)

Well, that you start thinking a little differently about things maybe, that maybe you can accept things, and that maybe there are still solutions to some things. (Interview X1)

Some hoped to integrate or process life-events and feelings:

I also didn’t want to keep that anger, because I want to, I’m not that far yet, but I want to get on with my life at some point. But that means you have to process things. (Interview X5)

Others were looking for a step further, they wanted to develop a grip on life, and/or find a future perspective:

I hoped to find someone who could help me regain peace of mind, to regain a grip on essential areas of life that slip through your fingers when you have to deal with earthquakes for such a long time on your own. (Interview X2)

Some clients wanted to connect more with themselves, others, and/or the sacred:

That you learn to love yourself, those are abstract phrases for me. (Interview X20).

Lastly, regarding the third domain of well-being, clients hoped to feel better and/or find peace.

Yes, that I could have a good talk with her and that maybe I would start to feel a little better. That was actually the goal. (Interview X1).

Some clients did not hope for anything specific.

### Experienced Outcomes

Clients reported many different outcomes. They were articulated on the same 3 domains as the goals of care, with 9 subdomains.

#### The Relationship with the Chaplain

Regarding the first domain, clients firstly report being seen, heard, and acknowledged. This included having been able to tell their story in a respectful conversation:

We’re kind of on the same wavelength and we understand each other. We don’t need so many words to understand each other and that makes a big difference. Because in this world, if you’re among the unhoused, yes, most of them are under influence and are often on a different level, and then you have the care organizations who think in completely different directions. And then it’s nice when you have someone, yes, who can think on the same level. (Interview X16)

It also included being seen as a human being instead of a number:

Well, first of all, what I found refreshing and surprising, was that I didn’t feel like I was a patient or a client or a customer, [. . .], I haven’t felt like a number. (Interview X2)

It finally included feeling that you could be yourself, and that you could tell everything without judgment from the chaplain:

She makes me feel not crazy. [. . .] she knows you’re just being damaged. It’s not your fault. (Interview X3)

Second, clients felt (temporarily) less lonely because of the relationship with the chaplain:

Because I’ve been living here on the street for five years, I’ve established a bond with her. And well, the bond has continued and I, when I come here, I still feel at home. (Interview X16)

Third, clients had regained trust through their relationship with the chaplain. For some, this was just trust in the chaplain, others also trusted themselves, other people and/or the government more. Part of this had to do with the fact that the chaplain had become an important part of clients’ support system, which made them feel reassured and safe. Clients described the chaplain as “a lifebuoy,” “kept me on my feet,” “has been my rescue,” and “a footing” (e.g., within the dynamic life of the unhoused).

We have trust issues because of all that has happened, and it has been a steady build up to trust, just really through the trust and the support she gives us. (Interview X3)

#### Meaning-Making

Regarding the second main domain, the meaning-making of the client, clients first reported a better understanding of themselves, others, and the broader context. They were able to give words to what was happening with them, see things from another perspective, and contextualize life-events:

Maybe I can put it more concretely, that in the treatment they really [. . .] start asking questions: where were you standing? What did you see? What did you smell? Just say all those kinds of details. Very much zooming in. With the chaplain it was more about: with whom were you there on deployment? And how were you trained in the past? [. . .] Then I found out, for example, that my entire instructor group [. . .] when I was trained at seventeen consisted entirely of veterans. Well, that had, in retrospect with the knowledge of today, quite an impact on how I was trained. To see the very big picture, allowed me to understand much better of what all happens on a deployment like that. (Interview X14)

Second, clients reported they can allow themselves to feel their own emotions and let go of them. They were able to “find meaning in,” “accept,” “find peace with,” “come to terms with,” and/ or “process” life-events:

And with all the emotions that passed, she remained present and nearby [. . .]. Such closeness, if you can give that to another person, that feeling, only then can you slowly begin to feel safe again. Yes, and only then can you turn your gaze deeper inward and look at what grieves you most deeply. (Interview X2)

Third, as a next step, clients reported that they had gained a better grip on life. This doesn’t necessarily mean that life circumstances have changed or are solved, but that clients are able to bear them:

The chaplain can’t influence anything around my demolition-new construction process, but she can shape with me how I’m doing, and because of that I can bear this. Otherwise, I wouldn’t have been able to bear this, even in the coming time. (Interview X2)

Fourth, clients described that they saw a path forward, that they had regained a future perspective. They experienced renewed hope, a sense of balance. The chaplain had empowered them. Some clients translated this into concrete actions such as going outside of their home, restarting work, going on a trip, or joining a (sports) peer group:

I thought: well, there’s hope for the future. I thought: there’s hope. (Interview X6)

Fifth, clients spoke about a regained connection with themselves. They reported increased self-worth, being able to choose for themselves, and having gotten closer to themselves. Moreover, they described a regained connection with others, and (sacred) sources of strength:

Well, I have come a whole lot closer to myself again. I really do feel like I have my feet just a little bit more back on the ground. At one point you feel like such a punching bag, being pushed back and forth by all kinds of agencies. (Interview X5)

#### Well-Being

Finally, with regard to the third main domain, clients reported increased well-being. This comprised “the heaviness is gone,” the “burden is off the shoulders,” “feeling lighter,” being “less gloomy,” and “the future is sunnier.” It also included feeling “relaxed,” “relieved,” “more space,” “content,” “more comfortable in your own skin,” “cheerful,” “uplifted,” “happy,” “energized,” “reborn,” “hopeful,” and “peaceful.”

For 2 clients, for whom there was a mismatch between their existential concerns and the amount of chaplaincy care, there were no substantial outcomes to report:

Yes, because there is or was too little contact with the chaplain, you can’t really explore certain things further. You can talk about your problems, but then the time is too short to elaborate. Then it stays in your head. (Interview X1)

### Effective Elements

As effective elements that lead to these outcomes, clients mentioned 3 domains. First, they spoke about the relational qualities of the chaplain. The chaplain listened well without judgment, time-pressure or an agenda; made space for the development of a reciprocal trusting relationship; did not abandon them, was always available, and sometimes did something extraordinary such as joining a visit to the lawyer. Qualities of the chaplain included openness, honesty, clarity, empathy, analytic capabilities, a good memory, and a bridge to the divine. Second, they spoke about the meaning-making competencies of the chaplain. The chaplain helped them reflect by asking good questions, putting life-events into a larger perspective and/or including their relational context, and by working with creative embodied practices in connection to the life-world of the client. Furthermore, the chaplain wasn’t afraid of facing emotions; gave non-binding advice; and empowered clients. Some pointed out that the chaplain acted as an intermediary between different contexts and could build bridges between them for the benefit of the client. Finally, the chaplain did not go along with the dominant (organizational) narratives, and offered a sanctuary, a safe space, through chaplains’ confidentiality and by visiting people at home.

## Discussion

In response to the first part of the research question “What are primary and community care clients’ reasons to seek chaplaincy care?,” we found that clients sought support from the chaplain for existential concerns and intense existential struggle. These included experiences of loss, loneliness and fear of death. The results overlap for the most part with results from the 3 other studies conducted in this context.^22,26,30^ Also there, a significant number of life events has “knocked them [clients] sideways” and eroded their sense of self-efficacy and security in life.^30^ The studies mention loss of hope/meaning in life, isolation, fear of death, concern for family, and shame/guilt as main concerns. They also list support with medical decisions, a concern absent from our study, possibly because chaplains’ role herein has not been emphasized.^40^ Finally, they list religious/spiritual concerns and doubts about faith, possibly not mentioned in this study because of the secular context of The Netherlands. More research into clients’ reasons, particularly of a diversity of clients that was not seen in the previous studies, is important to ensure that chaplains in primary and community care settings reach those people who are in need of chaplaincy care.

In response to the second part of the research question “What are primary and community care clients’ ideas of care goals?,” clients formulated goals in 3 domains: a good relationship with the chaplain, a next step in their meaning-making, and improved well-being. We are not aware of previous studies into the goals of care of clients. A study about chaplaincy’s goals according to researchers and chaplains, however, shows similar domains, such as support towards relational affirmation, worldview vitality and plausibility (meaning making), processed life events (meaning-making), deepened spirituality, and improved well-being.^41^ Clients’ and chaplains’ goals seem well-aligned. This suggests that the primary and community care clients involved in our study have an accurate idea of what to expect from a chaplain. There might be different reasons for this, partly depending on the type of clients involved. For instance, veterans might know what chaplaincy entails from the time that they were active in the military; we have seen that unhoused people know the chaplain who works in their specific neighborhood through chance encounters; and for some types of clients there was a good referral system and regular consultation between chaplains and other professionals. Also, we see that outpatient chaplaincy goals largely overlap with inpatient chaplaincy goals. We are now ready to take the next step around goals research, for example to explore whether there is a difference in emphasis on certain chaplaincy goals across distinct settings. Some work has already been done around this theme, see Rosie et al.^42^

In response to the third part of the research question “What outcomes of care do clients’ report?,” outcomes are mentioned within the same 3 domains as goals: the good relationship with the chaplain led to feeling seen, heard, acknowledged, less lonely, and more trusting. Support with meaning-making amounted to insight, processed emotions and life-events, grip on life, a future perspective, and regained connections. Finally, regarding well-being, clients reported feeling more positive and peaceful. In the one other qualitative study into the perceived impact of chaplains in primary care in Scotland, clients felt that the chaplain preserved their dignity by an affirming relationship. Through this, clients acquired the courage and strength to face their concerns and struggles and rebuild self-confidence and resilience. They were able to re-engage in life with meaning and purpose.^30^ We see a similar pathway in our results, but the clients in our study did not all reach the “end” of the pathway—they also reported meaningful outcomes “along the way.” Finally, some quantitative studies into chaplaincy outcomes in primary and community care report higher (spiritual) wellbeing, higher (health-related) quality of life, and better religious and general coping.^16,23,31-34^ The qualitative results of this study might provide insight into what aspects mediate these quantitative outcomes.

For most clients, their reasons to seek chaplaincy care, ideas for care goals, and experienced outcomes are in line with each other. This may be influenced by selection bias, see the limitations section, but surely a selection of chaplaincy clients assess their process positively. How do we weigh those findings? Does this automatically mean that we can speak of “good chaplaincy care”? Are reasons and goals, such as the alleviation of loneliness, part of what we aspire to as chaplains? One might argue that the (cultural) dominance of individualized spirituality erodes meaningful (r/s) communities and networks.^43^ Chaplains in primary and community care possibly fill this gap. This, however, raises the question of whether chaplains, by focusing on one-on-one conversations, reinforce the dominance of individualized views of spirituality. What if we would understand primary and community care chaplaincy as not predominantly aiming to provide support on an individual level, but also as doing political work in public space?^44^ This would involve a shift in focus toward goals such as fostering meaningful communities and initiating public dialogue about how dominant societal visions impact people’s capacity for meaning-making.

Finally, we can ask questions about the relationship between the client and the chaplain. Some clients in our findings express their strong dependency on the chaplain in being seen and heard, in re-engaging life. Others note the importance of the fluid length and forms of their contact with the chaplain. Is there an endpoint in chaplaincy care, a moment when chaplaincy’s goals of care are reached? Do we train our chaplain’s in boundary setting, such as the ability to complete relationships?^45^ Regarding the distribution of health care finances in times of scarcity, are 5 conversations enough or should long-term care be the standard? We warrant continuous reflection on these questions.

### Limitations and Future Research

Firstly, this study was limited to chaplaincy care in one-to-one contacts. To develop a more comprehensive overview of spiritual needs, goals, and outcomes of chaplaincy care for primary and community care patients, future research should also examine other levels of chaplaincy care such as group support, rituals, the education of other (informal) healthcare professionals, and organizational- and policy-related activities.

Second, the respondents were recruited by the chaplains themselves. Although they invited all clients they had seen in the past 3 months, a selection bias might have occurred. Also, due to self-reporting of respondents, social desirability bias may have been present. Most of the respondents were very positive about the chaplaincy care they received. Only 2 respondents were critical, due to the limited number of visits of the chaplain. These biases in the interviews might have influenced the findings. Future research could try to select a more random sample and mitigate desirability bias by triangulating data sources.

Third, respondents were predominantly supported by Christian and Humanist chaplains, and were primarily Christian and unaffiliated themselves. There was an underrepresentation of chaplains and respondents from other worldview traditions. Finally, we did not manage to include all chaplaincy working areas in primary and community care, such as their support for people with mental disabilities or parents with palliative children. Future research could reach out to these areas as well.

## Concluding Remark

In this study we sought to extend to extend the evidence-base for chaplaincy in primary and community care by focusing on the perspective of chaplaincy clients. The findings indicate that clients feel supported through the relationship with the chaplain, in their meaning-making, and experience an increase in their well-being. This indicates that chaplains might bring an important new perspective to the healthcare landscape of primary and community care.

## Notes

i. In the Netherlands, chaplains in primary and community care are financed through various modes of funding. For the 50-plus year old and the mental health clients, the government subsidizes chaplaincy care up to 5 conversations for people above 50 years and palliative patients regardless of their age (clients younger than 50 years have to pay for chaplaincy care themselves, this group however was not part of the study). Chaplaincy care for veterans is paid for by the ministry of defense without a limit on the number of conversations. Chaplaincy care for the victims of gas extraction is financed through the ministry of economic affairs, also without a limit on the number of conversations. Chaplaincy care for the unhoused is financed through various subsidies and donations.
